# Marital Status and Sociodemographic and Clinical Correlates in Patients With Mental Disorders in Northwestern Nigeria

**DOI:** 10.7759/cureus.107067

**Published:** 2026-04-14

**Authors:** Abdullahi Ibrahim, Anas I Yakubu, Baguda S Abubakar, Abdulfatai T Bakare, Abubakar Ahmad, Abbas A Yusuf, Gidado M Ibrahim, Bioku A Abass, Eliyah N Nwabunwanne, Fawaz Babandi

**Affiliations:** 1 Psychiatry, Federal Neuropsychiatric Hospital, Sokoto, NGA; 2 Psychiatry and Behavioral Sciences, Federal Teaching Hospital, Birnin Kebbi, NGA; 3 Psychiatry, Usmanu Danfodiyo University Teaching Hospital, Sokoto, NGA; 4 Psychiatry, National Hospital Abuja, Abuja, NGA; 5 Psychiatry, Federal Neuropsychiatric Hospital, Kaduna, NGA; 6 Psychiatry, Royal Hobart Hospital, Hobart, AUS; 7 Family Medicine, Usmanu Danfodiyo University Teaching Hospital, Sokoto, NGA; 8 Psychiatry, Abubakar Tafawa Balewa University Teaching Hospital, Bauchi, NGA

**Keywords:** divorce, marriage, mental illness, nigeria, unmarried

## Abstract

Background: Marriage is one of the most important life events for an individual because it can influence psychological well-being and social functioning and, in some communities, has been linked to quality of life. Yet, for several reasons, individuals with mental illness have difficulty contracting and/or maintaining a successful marriage. This study aimed to determine the marital status and sociodemographic and clinical correlates among patients with mental illness in northwestern Nigeria.

Materials and methods: A cross-sectional study was conducted at the medical outpatient department of Usmanu Danfodiyo University Teaching Hospital, Sokoto (UDUTHS), among patients with mental illnesses attending routine follow-up visits over a three-month period from October 2025 to December 2025. A total of 226 participants were recruited using a consecutive sampling technique after obtaining informed consent. A structured questionnaire was used to collect sociodemographic information, such as marital status, and clinical data, such as diagnoses. Data analysis was performed using SPSS Statistics version 25.0 (IBM Corp. Released 2017. IBM SPSS Statistics for Windows, Version 25.0. Armonk, NY: IBM Corp.). Descriptive statistics were used to summarize the data, and associations were assessed using the chi-square test.

Results: The mean age of the participants was 31.7 ± 10.9 years, and most were aged 25-44 years. Most participants married at a younger age, whereas the onset of illness occurred at an older age. A slight majority (50.4%) were married, and most marriages occurred before the onset of illness; the rate of divorce was much higher after illness onset. Mood and substance use disorders were the most common diagnoses among the respondents. There was a significant association between marital status and age category (p = 0.019), age at illness onset (p < 0.001), sex (p < 0.001), level of education (p = 0.015), consanguinity (p = 0.001), marital discord (p = 0.012), diagnosis (p < 0.001), with mood disorders having the highest unmarried status, timing of divorce (p = 0.007), and timing of marriage (p = 0.007).

Conclusions: This study revealed that psychiatric disorders play an important role in the matrimonial outcomes of individuals with mental illness. The findings indicate that individuals with mental illness predominantly experience divorce following the onset of their illness. Several factors play key roles, including advanced age, female sex, low level of education, diagnosis (particularly mood and substance use disorders), and marital discord. Family-based interventions and marital counseling should be routinely incorporated into mental health clinics, along with psychological interventions for family members to address the impact of stigma on married and unmarried individuals with mental illness.

## Introduction

Marriage can be defined as a union between two individuals to fulfill cultural expectations, such as intimacy and procreation [1]. It is one of the most important life events, as it reflects levels of maturity, social status, societal acceptance, and social support [2]. An individual's psychological well-being and social functioning can be influenced by marriage [3]. In some communities, marriage has also been linked to an individual's quality of life [1].

For individuals with a major mental illness, marriage can be a protective factor, as being in a stable marital union has multiple benefits and advantages [4]. These include enhanced social support, social integration, companionship, life satisfaction, reduced stigma associated with being unmarried, less psychological distress, and improved overall health [1,5]. The benefits extend beyond mental health, as marriage improves not only psychological adjustment but also physical health [6]. Furthermore, encouragement from spouses has been shown to improve adherence to pharmacotherapeutic advice and to lead to more regular clinic visits [7]. Parenting roles and other family responsibilities, which may precipitate or perpetuate mental illness, can also be made less demanding within a supportive marriage [1].

One outcome of a successful marriage is having children. Although parenthood comes with many responsibilities, when children are properly raised, they may contribute positively to the mental stability of individuals with mental illness. For example, children may provide parents with a sense of fulfillment and meaning in life, greater optimism and hope for the future through their children's aspirations, and motivation to adopt healthier behaviors, such as ceasing the use of psychoactive substances [8,9]. Even trust in mental healthcare providers has been reported to be greater among parents with mental illness than among those without children [8].

Despite the considerable benefits of marriage for people with or without mental illness, individuals with major mental illness often have difficulty getting married because of challenges in forming and maintaining relationships that may lead to marriage [10]. Contributing factors include stigma, disclosure-related concerns, negative societal attitudes, financial instability, unemployment, low socioeconomic status, and severe symptoms such as psychosis [1,11]. Impairments in social functioning, such as social withdrawal, which are common features of some major mental illnesses, may also pose substantial barriers to marriage [12].

Among the relatively few married individuals with mental illness, the rates of divorce and marital dissolution have been reported to be high [13,14]. Compared with the general population, the risk of divorce is 20-80% higher [14]. For example, approximately four out of 10 patients with schizophrenia experience marital dissolution or separation [1].

In northern Nigeria, marriage is a common practice and is largely shaped by cultural and religious beliefs. However, the literature on marital patterns and their associated factors in this region remains limited. Therefore, this study may provide a foundation for future research in this important area. Specifically, the study aims to determine marital status and evaluate its association with sociodemographic characteristics, including sex, age group, age at marriage, age at onset of illness, educational attainment, employment, and income, as well as clinical variables such as consanguinity of marriage, marital discord, divorce, and diagnosis among patients with mental illness in northwestern Nigeria.

## Materials and methods

This cross-sectional study was conducted at the psychiatric outpatient department of Usmanu Danfodiyo University Teaching Hospital, Sokoto (UDUTHS), among patients with mental illnesses attending routine follow-up visits. The study period spanned three months, from October 2025 to December 2025.

Eligibility criteria

The study population comprised patients attending routine follow-up care at the psychiatric outpatient clinic of UDUTHS during the study period. Inclusion criteria were patients aged 18 years or older, patients diagnosed with a psychiatric disorder by a consultant psychiatrist, and patients who provided informed consent. Exclusion criteria were patients who did not provide informed consent, those with severe physical or mental disorders that hindered participation, and participants whose marital history could not be reliably obtained, particularly when confirmation or corroboration by a significant other was not possible.

Sample size and sampling technique

All patients attending routine follow-up visits during the three-month study period were consecutively enrolled. A total of 226 eligible participants were recruited, ensuring the inclusion of all consenting and accessible patients.

Data collection procedure and instruments

The questionnaire used in this study was developed by the authors in accordance with the study objectives and previous literature, using the biopsychosocial framework. Key variables included sex, age, marital status, family type, and clinical diagnosis, as reported in earlier studies. The questionnaire consisted of three sections: sociodemographic, marital, and clinical characteristics. It was reviewed by the authors and pretested with 26 subjects who were not included in the main study, resulting in modifications to some sections.

The questionnaire was designed as a structured, interviewer-administered instrument consisting primarily of closed-ended questions to enhance consistency and facilitate analysis. Patients' diagnoses were established by the attending consultant psychiatrist using the ICD-11 as the diagnostic framework. Accordingly, the diagnoses provided by the psychiatrists were adopted for this study. The researchers were not involved in the diagnostic process, which remained the responsibility of the primary managing psychiatrist. The final version of the questionnaire is included in the appendices.

Data management and statistical analysis

Data were collected, double-checked, entered, and analyzed using SPSS Statistics version 25.0 (IBM Corp. Released 2017. IBM SPSS Statistics for Windows, Version 25.0. Armonk, NY: IBM Corp.). Descriptive statistics, including frequencies, percentages, and means with standard deviations, were used to summarize the data. At the bivariate level, the chi-square test was used to assess associations between marital status and sociodemographic and clinical variables. Statistical significance was set at p < 0.05.

Ethical consideration

Ethical approval was obtained from the Usmanu Danfodiyo University Teaching Hospital Health Research Ethics Committee (approval number: UDUTH/HREC/2026/1717/V1).

## Results

A total of 226 patients attending routine follow-up at the psychiatric outpatient clinic were recruited.

Distribution of the sample by sociodemographic variables

Table 1 shows that the respondents had a mean age of 31.7 ± 10.9 years. Most were aged 25-44 years, were female, belonged to the Hausa ethnic group, and practiced Islam. The majority had education beyond the secondary level and came from polygynous families. Participants generally married at a younger age, whereas illness onset typically occurred in older individuals. A slightly larger proportion of respondents were married. Of note, the data on age at marriage indicate that 124 individuals had ever been married. However, current marital status shows that 114 respondents were currently married (including those who were separated), with the difference reflecting individuals who were divorced. Thus, 124 represents the number of respondents who had ever been married, while 114 represents those who were currently married.

**Table 1:** Distribution of the sample by sociodemographic variables

Variable	Frequency	Percentage
Age category		
<25	58	25.7
25-44	136	60.2
>45	32	14.2
Marital status (dichotomized)		
Married	114	50.4
Unmarried	112	49.6
Age at marriage (n = 124) 21.8 ± 5.5 years		
<22	100	80.6
≥22	24	19.4
Age at onset of Illness 26.2 ± 9.7 years		
<26	28	12.4
≥26	198	87.6
Gender		
Male	78	34.5
Female	148	65.5
Tribe		
Hausa	194	85.8
Yoruba	8	3.5
Igbo	8	3.5
Others	16	7.1
Religion		
Islam	210	92.9
Christianity	16	7.1
Level of education		
Secondary and below	98	43.4
Above secondary	128	56.6
Family type (parents)		
Monogamous	92	40.7
Polygamous	134	59.3

Distribution of the marital status among unmarried respondents

**Figure 1:**
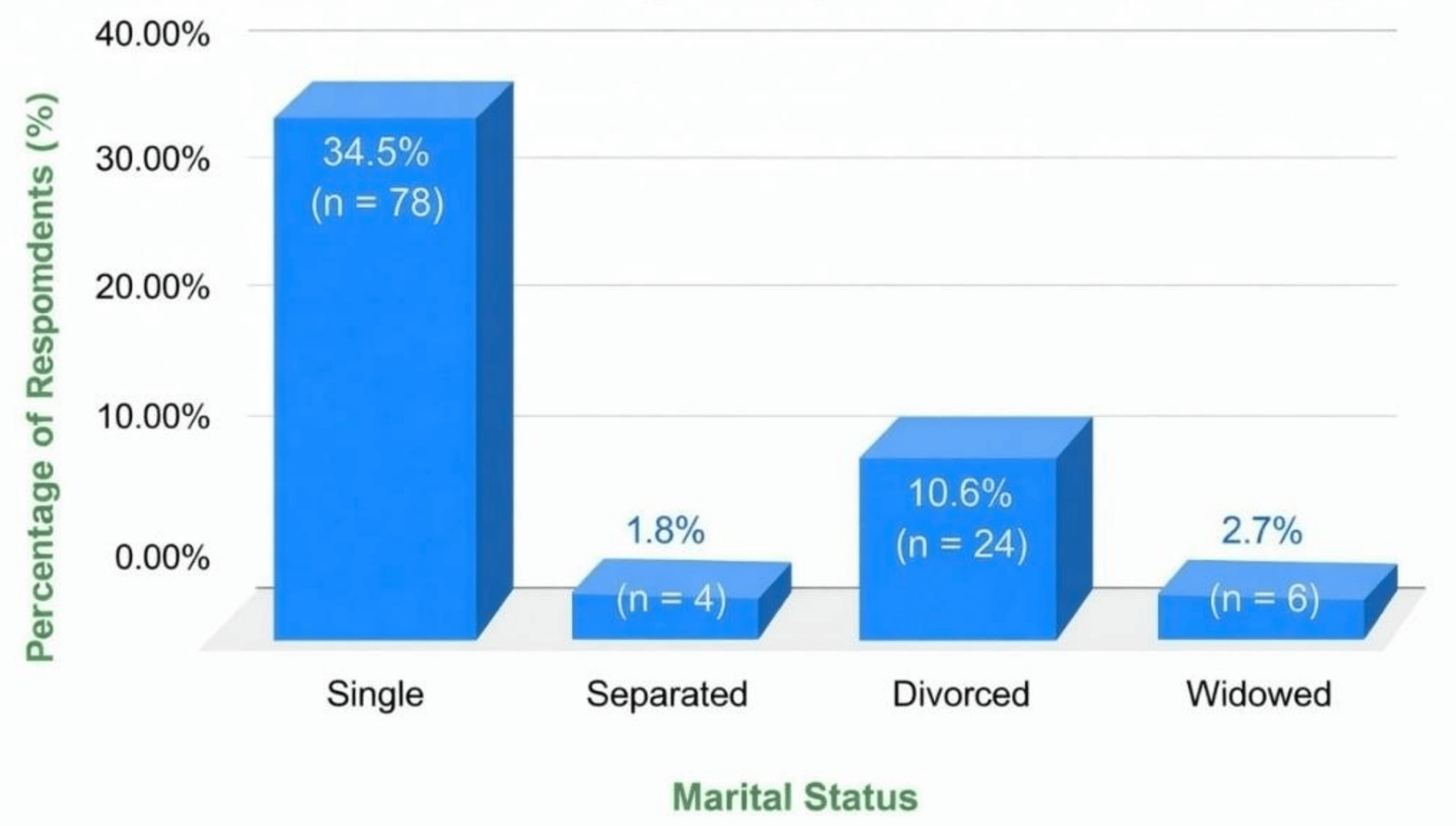
Distribution of the marital status among unmarried respondents

Figure 1 illustrates the distribution of marital status among unmarried participants, with the majority reporting never being married (single).

Clinical and marital characteristics of the respondents

Table 2 presents the clinical and marital characteristics of the respondents. It shows that most marriages occurred before the onset of illness. Although marital discord was more commonly reported before illness onset, the rate of divorce was much higher after the onset of illness. Furthermore, most marriages were non-consanguineous, and participants were predominantly in monogamous unions. Mood and substance use disorders were the most common diagnoses among the respondents.

**Table 2:** Clinical and marital characteristics of the respondents

Variable	Frequency	Percentage
If divorced, when? (n = 18)		
Before illness	2	11.1
After illness	16	88.9
Consanguinity (n = 124)		
Yes	16	13.3
No	108	86.7
If married, when? (n = 124)		
Before illness	97	78.5
After illness	27	21.5
Marital discord (n = 124)		
Yes	32	25.7
No	92	74.3
If there is marital discord when (n = 48)		
Before illness	26	54.2
After illness	22	45.8
Family type (parents) (n = 226)		
Monogamous	92	40.7
Polygamous	134	59.3
Family type (self) (n = 124)		
Monogamous	90	72.3
Polygamous	34	27.7
Diagnosis category		
Psychotic disorders	58	25.7
Mood disorders	80	35.4
Others	2	0.9
Substance use disorders	86	38.1

Factors associated with marital status

Table 3 summarizes the factors associated with marital status, including sociodemographic, marital, and clinical variables. There was a significant association between marital status and age category (p = 0.019), with the highest proportion of unmarried participants observed among those aged over 45 years. Similarly, age at onset of illness was significantly associated with marital status (p < 0.001), with early onset being more common among unmarried individuals. Sex was also significantly associated (p < 0.001) with a higher proportion of unmarried females. Level of education showed a significant association (p = 0.015), with a higher proportion of unmarried status among those with lower educational attainment. Consanguinity was significantly associated (p = 0.001) with a higher proportion of non-consanguineous marriages among unmarried participants. Marital discord was significantly associated with marital status (p = 0.006), with a higher proportion among those reporting marital discord. Diagnosis was also significantly associated (p < 0.001), with mood disorders showing the highest proportion of unmarried patients.

**Table 3:** Factors associated with marital status p < 0.05, * Fisher's exact test

Variable	Unmarried n (%)	Married n (%)	χ²	df	p-value
Age category (years)			7.98	2	0.019
<25	22 (37.9%)	36 (62.1%)			
25-44	70 (51.5%)	66 (48.5%)			
>45	22 (68.8%)	10 (31.2%)			
Age at onset (years)			23.97	1	<0.001
<26	2 (7.1%)	26 (92.9%)			
≥26	112 (56.6%)	86 (43.4%)			
Age at marriage (n = 124)			0.100	1	0.788
<22	78 (78.0%)	22 (22.0%)			
≥22	18 (75.0%)	6 (25.0%)			
Sex			23.56	1	<0.001
Male	32 (28.2%)	73 (71.8%)			
Female	82 (62.2%)	39 (37.8%)			
Level of education			*		0.015
Low education	65 (59.2%)	47 (40.8%)			
High education	49 (43.8%)	65 (56.2%)			
Employment status			*		0.087
Employed	50 (43.6%)	62 (56.4%)			
Unemployed	64 (54.1%)	50 (45.9%)			
Tribe			*		0.274
Hausa	56 (49.5%)	56 (50.5%)			
Yoruba	30 (75.0%)	10 (25.0%)			
Igbo	14 (25.0%)	30 (75.0%)			
Others	14 (50.0%)	16 (50.0%)			
Consanguineous marriage (n = 124)			12.09	1	0.001
Yes	6 (20.0%)	24 (80.0%)			
No	51 (54.1%)	43 (45.9%)			
Marital discord (n = 124)			*		0.006
Present	38 (65.5%)	20 (34.5%)			
Absent	30 (45.2%)	36 (54.8%)			
Diagnosis category			28.46	3	<0.001
Psychotic disorders	26 (44.8%)	32 (55.2%)			
Mood disorders	40 (67.5%)	20 (32.5%)			
Others	48 (48.6%)	42 (51.4%)			
Substance use disorders	0 (0.0%)	18 (100.0%)			
Divorce timing (if divorced when) (n = 18)			*		0.007
Before illness	8 (100%)	0 (0.0%)			
After illness	0 (0.0%)	10 (100.0%)			
Marriage timing (if married when) (n = 124)			7.87	1	0.007
Before illness	70 (72.6%)	26 (24.7%)			
After illness	13 (47.1%)	15 (52.9%)			

Furthermore, there was a significant association between the timing of divorce and marital status (p = 0.007), with divorce occurring more frequently after the onset of illness. There was also a significant association between timing of marriage and marital status (p = 0.007), with marriages more likely to have occurred before the onset of illness.

## Discussion

The current study aimed to investigate patterns of marital status and associated factors among patients with mental illness in northwestern Nigeria. The sociodemographic findings showed that most respondents were between 25 and 44 years of age, female, and Hausa Muslims, reflecting the cultural composition of this region of Nigeria. This is consistent with a previous study conducted in the same geographical area [15]. Furthermore, the mean age of respondents in this study (31.7 ± 10.9 years) indicates a young-to-middle-aged adult population. This age range coincides with the typical onset period of major mental illnesses (approximately 60% of cases begin before 25 years of age) as well as the period during which individuals commonly enter marriage. For instance, mood disorders and substance use disorders often begin during this life stage, which is also a key social milestone for marriage and family formation [16-18]. The study indicates that approximately 50% of individuals with mental illness are married, which is lower than the marital prevalence in the general Nigerian population (approximately 86%). Recent studies have also shown that marriage remains highly prevalent in Nigeria [19,20].

Findings from various studies have reported gender differences in marital status among patients with major psychiatric disorders [21,22]. This study is no exception, as it demonstrated a significant association between gender and unmarried status, with female respondents showing a higher proportion of unmarried individuals than male respondents. This is similar to findings from Ethiopia, where men with major mental illness have a higher likelihood of contracting marriage than their female counterparts [2]. This disparity has been attributed to the disproportionate stigmatization of women with mental illness, leading to discrimination in marriage [23].

Although most respondents had attained higher levels of education, the study found a significant association between education and marital status, with lower educational attainment being more common among unmarried individuals. This finding is consistent with Choi et al., who reported that low educational attainment and unmarried status among individuals with mental illness frequently co-occur and may even increase the risk of suicide [24]. Higher education is generally associated with improved employment opportunities, better social functioning, and higher socioeconomic status, all of which are important determinants of successful marriage [4].

The present study also revealed that most individuals with mental illness entered marriage before the onset of illness, while most divorces occurred after illness onset. Similar findings have been reported in previous studies [4,13,14]. Mood disorders and substance use disorders were identified as the most prevalent diagnoses among unmarried individuals. Similarly, Zha et al. reported that unmarried individuals exhibit significantly higher levels of depressive symptoms compared with married individuals [25]. Furthermore, residual symptoms of mood disorders, such as fatigue, anhedonia, and loss of interest, which often persist after remission, may pose additional challenges to maintaining stable marital relationships. These symptoms can impair communication, reduce emotional reciprocity, and compromise intimacy, thereby contributing to marital instability and reduced satisfaction [13].

Additionally, behavioral dysregulation, reduced emotional availability, and occasional aggression among individuals with substance use disorders may contribute to marital dissatisfaction and dissolution [26]. Recurrent relapses and cycles of rehabilitation may further strain relationships, making marital cohesion difficult to sustain [27]. These disorders are widely recognized as being associated with significant interpersonal difficulties, including relationship instability and marital breakdown [14]. The impact of marital distress associated with mood and substance use disorders may be further amplified in the Hausa community, given its strong emphasis on social and cultural institutions, particularly marriage and its associated expectations [28].

The present study has several limitations. Its cross-sectional design limits the ability to establish causal relationships between sociodemographic and clinical factors and marital status. Additionally, recall and reporting biases may have occurred, as data were obtained through self-reports from patients or significant others. Furthermore, the single-center design limits the generalizability of the findings beyond the study setting.

Future multicenter and community-based longitudinal or prospective studies are recommended to better characterize marital patterns across different regions and populations. Such studies would also help clarify causal relationships and the challenges associated with marriage among individuals with mental illness. Given the high rate of divorce observed, family-based interventions and marital counseling should be routinely incorporated into mental health services, along with psychosocial interventions for family members to address the impact of stigma on both married and unmarried individuals with mental illness. Community awareness campaigns may also help reduce stigma and improve understanding of mental illness and its management, thereby addressing misconceptions about this vulnerable population.

## Conclusions

This study demonstrated that psychiatric disorders substantially influence the marital outcomes of individuals with mental illness. It revealed that most individuals with mental illness are married prior to the onset of their condition. Although marital issues may precede the onset of illness, most divorces occur after the development of the illness.

The study also identified several sociodemographic and clinical factors associated with a higher likelihood of remaining unmarried. These include advanced age, female sex, lower educational attainment, diagnoses such as mood and substance use disorders, and marital discord. These factors are likely to affect social functioning, increase stigma, and impair interpersonal relationships, thereby negatively influencing both relationship formation and stability. In particular, marital discord emerged as a key relational factor, underscoring the importance of relationship quality in determining marital outcomes among individuals with mental illness.

Finally, the findings highlight the importance of integrated, culturally sensitive, and family-oriented interventions in addressing both clinical and relational challenges and improving interpersonal functioning among individuals with mental illness, with the ultimate aim of enhancing marital stability and overall psychological well-being in the wider population.

## Appendices

**Table 4:** Study instrument

Section	Question	Response options
Section A: sociodemographic characteristics		
1	What is your age (in years)?	________
2	Which of the following age groups do you belong to?	☐ <25 years ☐ 25–44 years ☐ ≥45 years
3	What is your sex?	☐ Male ☐ Female
4	What is your tribe/ethnicity?	☐ Hausa ☐ Yoruba ☐ Igbo ☐ Others (specify): ______
5	What is your religion?	☐ Islam ☐ Christianity ☐ Others (specify): ______
6	What is your highest level of education?	☐ Secondary and below ☐ Above secondary
7	What is your current employment status?	☐ Employed ☐ Unemployed
8	What type of family were you raised in?	☐ Monogamous ☐ Polygamous
Section B: marital characteristics		
9	What is your current marital status?	☐ Married ☐ Unmarried
10	If unmarried, what is your marital status?	☐ Single ☐ Divorced ☐ Separated ☐ Widowed
11	At what age did you first get married (in years)?	________
12	Did your marriage occur before or after illness onset?	☐ Before illness ☐ After illness
13	Is your marriage consanguineous?	☐ Yes ☐ No
14	What type of family do you currently have?	☐ Monogamous ☐ Polygamous
15	Have you experienced marital conflict?	☐ Yes ☐ No
16	If yes, when did it occur?	☐ Before illness ☐ After illness
17	If divorced, when did it occur?	☐ Before illness ☐ After illness
18	What was your marital status before illness?	☐ Married ☐ Not married
Section C: clinical characteristics		
19	At what age did your illness start (in years)?	________
20	What diagnosis were you given by the doctor?	☐ Psychotic ☐ Mood ☐ Substance use ☐ Others (specify): ______
